# Integrating and rationalizing public healthcare services as a source of cost containment in times of economic crises

**DOI:** 10.1186/s13052-016-0231-1

**Published:** 2016-02-24

**Authors:** Massimo Pettoello-Mantovani, Leyla Namazova-Baranova, Jochen Ehrich

**Affiliations:** Institute of Pediatrics, University of Foggia, Via Pinto, 1, Foggia, 71100 Italy; European Paediatric Association, the Union of National European Paediatric Societies and Associations (EPA-UNEPSA), Berlin, Germany; Federal State Budgetary Institution “Scientific Centre of Children’s Health” Moscow, Russian Federation, Moscow, Russia; Children’s Hospital, Hannover Medical School, Hannover, Germany

**Keywords:** Public healthcare, Economy, Cost containment, Pediatrics, Children

## Abstract

**Background:**

Serious concern has been raised about the sustainability of public health care systems of European Nations and ultimately about the health of European citizens, as a result of the economic crisis that has distressed Europe since 2008. The severe economic crisis of the Euro zone, which is still afflicting Europe in 2016, has in fact threatened to equally impact public health services of nations presenting either a weak or a strong domestic growth.

**Comments:**

On behalf of the European Paediatric Association, the Union of National European Societies and Associations, the authors of the Commentary debates the relationship between the effects of economic instability and health, through the report on an article recently published in the Italian Journal of Pediatrics, which emphasized the importance of integrating existing public health care services, otherwise independently provided by public hospitals, and Primary Care Paediatric networks. The interconnections between the effects of economic instability and health are briefly commented, following the observation that these two factors are not yet fully understood, and that the definition of proper solutions to be applied in circumstances, where health is negatively impacted by periods of economic distress, is still open for discussion.

Furthermore it is noted that the pressure to “deliver more for less” often seems to be the driving force forging the political strategic decisions in the area of pediatric healthcare, rather than social, cultural, and economic sensitivity and competences. Thus, the delivery of appropriate pediatric healthcare seems not to be related exclusively to motivations aimed to the benefit of children, but more often to other intervening factors, including economic, and political rationales.

**Conclusions:**

The conclusions emphasize that local European experiences suggest that positive and cost effective healthcare programs are possible, and they could serve as a model in the development of effective cross-border regional program, not weakening the quality of services provided to children.

## Background

Serious concern has been raised about the sustainability of public health care systems of European Nations and ultimately about the health of European citizens, as a result of the economic crisis that has distressed Europe since 2008 [1]. The severe economic crisis of the Euro zone, which is still afflicting Europe in 2016, has in fact threatened to equally impact public health services of nations presenting either a weak or a strong domestic growth [1].

Although the relationship between the effects of economic instability and health has been the subject of a decades-long research engaging experts since the beginning of the last century, the interconnections between these two factors are not yet fully understood. The definition of problem solving solutions to be applied in circumstances where health is negatively impacted by periods of economic distress is still open for discussion [1–3].

The article recently published by Nigri et al. in the Italian Journal of Pediatrics [4] offers a substantially new and stimulating contribution to such an open debate, which is of great importance for the European public healthcare services. In fact, this paper describes the Pediatric Ambulatory Consulting Service (PACS) project, developed by an Italian regional Public Health Centers network, in response to the current general situation of economic distress. PACS integrates existing public health care services, otherwise independently provided by public hospitals and Primary Care Paediatric networks, with the purpose to establishing innovative yet efficient managerial solutions able to rationalize the resources, and not weakening the quality of services provided to the pediatric population. In brief it consists in a pediatric ambulatory consulting service, active in hospitals and providing a screening of the clinical conditions of outpatients <18 years old, before they access the Emergency Room (ER) Departments. The key question raised by the article is whether already existing local public health services can be further integrated in a cost-effective manner, while optimizing the efficiency and quality of the services offered to the population.

## The impact of globalization and economic changes on social and healthcare systems: need of innovative solutions

The well-known phenomena related to globalization have characterized economy and impacted social systems around the world during the last thirty years [5]. According to the International Monetary Fund [6], the volume of world trade has expanded by five-fold over a 30 years period from 1985 and 2015, and a progressive financial integration has characterized the last three decades. By the time of the 2008 crisis, global capital flows were more than threefold their level in 1995. Then, virtually all Nations faced the need to adapt their internal socio-economic structure, including public heath, to the new global context [7].

As emphasized by the Managing Director of the International Monetary Fund, Ms Christine Lagarde, in a recent speech delivered at the US Chamber of Commerce, in a world of increasing economic interconnections, the challenges are greater, but so too are the opportunities [7]. We may borrow such notion to adapt it to the matter of developing proper cost-effective public health programs at European level. The aim would be alleviating the increasing costs that have afflicted the various governments in such a sensitive area for the management of their local National budgets as public health.

Based on the principles enunciated by Mrs. Lagarde, a number of possible solutions could be conceived and explored. This would test the will of the single Nations and their administrators to develop cost effective solutions, while ensuring that the aim of balancing the budget would not affect basic quality standards for public health.

To such regard, the experiences related to successful projects and programs developed in several local European realities, as in the case of PACS [1], could be usefully taken in consideration and studied in order to evaluate whether they could be exported and re-proposed in different economic settings at European transnational level, while properly adapting to different social and cultural contexts. This approach could be considered the reverse of the original concept of “glocalization”, a portmanteau of globalization and localization. In fact, as first described in the late 1980s in articles published by Japanese economists in the Harvard Business Review [8], the concept of glocalization typically implicates that a product or a service is specifically adapted to each locality or culture in which it is sold or proposed. In our case, instead of “localizing the global” the proposed process would be to “globalize the local”, by using and adapting local programs of public health which have proved to be successful, as models to be re-proposed in different, yet comparable socio-economic contexts. The PACS program, developed locally in Italy, may in fact represent a good example of effective socio-economic and cost-effective local programs, which could be taken into consideration to be exported if properly adapted at European continental level.

The data in the article of Nigri et al. report a mean saving of 110.160 Euro per 1000 hospitalizations during 2014, based on 18 % reduction of hospitalizations. Therefore showing the economic effectiveness of PACS and suggesting that such project could be taken into consideration as a model to be further developed in different geographic areas in Italy and possibly beyond its National borders. The working hypothesis that local programs of public health could be expanded at continental level is supported by the existence of public health programs which have been active within the European context during the recent years [9]. To such regard the cross-border care programs represent an example of effective existing plans that have been activated at local level within trans frontier collaborative structures, well-known under the label of Euroregios [10], with the aim to rationalize and possibly reduce the economic burden of public health among European nations.

## Conclusions

Europe has been defined as a giant “natural laboratory” for health systems, and a great chance for countries to learn reciprocally [11]. In fact, the health systems of Europe currently represent the greatest collective commitment to health anywhere in the world. However, while nations are all trying to do similar things in the area of healthcare management, they do it in very different ways, often resisting to cooperative proposals and to learning across borders [11]. In this respect, any successful local project which has proven to be cost-effective [1] should be taken into consideration by policy-makers and should be further studied with a continental perspective, including the economic, legal and social implications, in order to assess whether and in case to what extent they could become part of future cross-border collaboration plans on Euregio level.

Healthcare significantly impacts the annual budget of the European nations. Furthermore there is an urgent need for efficient solutions to allow implementation of high cost innovative technology and medication. Great efforts have been made by national European pediatric societies to contrast local decisions taken by legislators that would have negatively impacted child healthcare. Such local efforts have been strongly supported by the European Paediatric Association, the Union of National European Pediatric Societies and Associations (EPA-UNESA) which represent its member national pediatric societies at European and at the International Pediatric Association-IPA level.

The pressure to “deliver more health for less money” often seems to be the main driving force forging the political strategic decisions in the area of pediatric healthcare, instead of promoting social, cultural, and economic sensitivity and competence. Thus, the delivery of appropriate pediatric healthcare seems not to be related exclusively to motivations aimed at the benefit of children, but more often at other intervening factors including mere economic and political rationale [12]. Local experiences such as the one reported by the PACS project [1], suggest that positive and cost effective healthcare programs are possible and that PACS could serve as a new model in the development of effective programs in other European nations.
